# Diez años del “Programa de evaluación de desempeño de laboratorios de inmunogenética” y su impacto en la “Red de donación y trasplantes”

**DOI:** 10.7705/biomedica.7589

**Published:** 2024-12-23

**Authors:** Yazmín Rocío Arias-Murillo, María Angélica Salinas-Nova, Yesith Guillermo Toloza-Pérez, Miguel Ángel Castro-Jiménez

**Affiliations:** 1 Grupo Red de Donación y Trasplantes, Instituto Nacional de Salud, Bogotá, D. C., Colombia Instituto Nacional de Salud Grupo Red de Donación y Trasplantes Instituto Nacional de Salud Bogotá, D. C. Colombia; 2 Programa de Entrenamiento en Epidemiología de Campo - FETP, Instituto Nacional de Salud, Bogotá, D. C., Colombia Instituto Nacional de Salud Programa de Entrenamiento en Epidemiología de Campo - FETP Instituto Nacional de Salud Bogotá, D. C. Colombia

**Keywords:** inmunología del trasplante, trasplante de órganos, control de calidad, pruebas de aptitud de laboratorios, inmunogenética, pruebas inmunológicas., Transplantation immunology, organ transplantation, quality control, laboratory proficiency testing, immunogenetics, immunologic tests.

## Abstract

**Introducción.:**

El uso de las pruebas inmunológicas antes del trasplante de órganos sólidos es fundamental para disminuir el riesgo de rechazo y las complicaciones de los trasplantes. Los sistemas de control de calidad de los laboratorios que las realizan son, por tanto, necesarios para la práctica clínica. El Instituto Nacional de Salud implemento el “Programa de evaluación externa del desempeño para laboratorios de inmunogenética de trasplantes” en el 2014.

**Objetivo.:**

Evaluar el desempeño de los laboratorios con base en los resultados de cinco pruebas inmunológicas para trasplantes en Colombia entre el 2014 y el 2023, según las directrices del “Programa de evaluación externa del desempeño para laboratorios de inmunogenética de trasplantes”.

**Materiales y métodos.:**

Se estudió el desempeño de los laboratorios mediante la evaluación de cinco pruebas inmunológicas para trasplante: clasificación del HLAde clase I y II, prueba cualitativa y prueba cuantitativa para el panel de anticuerpos reactivos, PRA (*Panel Reactive Antibodies*), pruebas para antígenos individuales y pruebas cruzadas.

Se recolectaron los datos de los informes de cada laboratorio. Con base en las comparaciones entre laboratorios, se calificó el desempeño de cada uno como «bueno», «aceptable» o «inaceptable» para cada prueba. Se calcularon proporciones y se hizo un análisis de valores predichos con intervalos de confianza del 95 %.

**Resultados.:**

El número de laboratorios participantes varió entre 5 y 12 para cada prueba. La categoría de desempeño «buena» fue menor en el primer año. El mejor resultado fue para la PRA cualitativa, calificada como «buena» en todos los laboratorios durante ocho años. En las pruebas de HLA (2014), PRA cualitativo (2017 y 2019), pruebas cruzadas (2019) y *single antigen* (2017 y 2019), el porcentaje de laboratorios con desempeño catalogado como «bueno» fue menor que el esperado.

**Conclusión.:**

El desempeño observado fue «bueno» en todos los laboratorios en el último trienio, excepto para dos de las cinco pruebas: el HLA y la PRA (*Panel Reactive Antibodies*) cuantitativa.

## Abstract (en)

**Introduction.:**

The use of immunological tests before solid organ transplantation is essential to reduce the risk of rejection and post-transplant complications. Therefore, quality control systems in laboratories performing them are necessary for clinical practice. The Colombian *Instituto Nacional de Salud* implemented the external evaluation program of transplant immunogenetics laboratory performance in 2014.

**Objective.:**

To evaluate the performance of the laboratories that carried out five of the immunological tests for transplants in Colombia between 2014 and 2023, according to information from the external evaluation program of transplant immunogenetics laboratory performance.

**Materials and methods.:**

We conducted a study of laboratory performance considering five immunological tests for transplantation: HLA, qualitative and quantitative PRA (Panel Reactive Antibodies), isolated antigen, and cross-matching tests. We collected data from reports of each laboratory. Based on the comparisons between laboratories, their performance was rated as “good”, “acceptable”, or “unacceptable” for each test. We calculated proportions and an analysis of predicted values with a 95% confidence interval.

**Results.:**

The number of participating laboratories varied between 5 and 12, depending on the test. The proportion of laboratories with “good” performance was lower in the first year. The best performance was for qualitative PRA, rated as good in all the laboratories for eight years. In HLA (2014), qualitative PRA (2017 and 2019), crossmatch tests (2019), and single antigen (2017 and 2019) tests, the laboratories had a lower percentage of “good” performance than expected.

**Conclusion.:**

“Good” performance was observed in all the laboratories in each test during the last three years, except for HLA and quantitative PRA.

La Red de Donación y Trasplantes del Instituto Nacional de Salud de Colombia intercambia órganos para trasplantes. Los órganos de los donantes se distribuyen en distintas instituciones prestadoras de servicios de salud trasplantadoras, según los criterios de asignación de órgano sólido y los resultados de las pruebas inmunológicas. En el 2014, el Instituto estableció estándares de calidad para los laboratorios que practican pruebas de inmunología para trasplantes ^1^.

Junto con la expedición de estos estándares, y producto de la necesidad del país, se puso en marcha el “Programa de evaluación externa del desempeño para laboratorios de inmunogenética de trasplantes”, como una estrategia para verificar la calidad, la precisión y la confiabilidad de los resultados ofrecidos por los laboratorios implicados en las pruebas previas y posteriores al trasplante, para donantes y receptores. Este programa se diseñó e implementó para contribuir a los propósitos de la Red de Donación y Trasplantes (contenidos en el Decreto 2493 de 2004) y estimar el cumplimiento de los estándares de calidad expedidos por la coordinación nacional de la misma. El programa de evaluación se alinea con los estándares internacionales propuestos por la *American Society for Histocompatibility and Immunogenetics* (ASH I) ^2^ y la *European Federation for Immunogenetics* (EFI) ^3^.

La relevancia de los programas de calidad, reconocidos a nivel global, radica en su capacidad para verificar la uniformidad de los resultados del laboratorio en contraste con los estándares consensuados nacionalmente, lo que garantiza la disponibilidad de información -fiable y comparable-de la Red de Donación y Trasplantes, sin importar la geografía.

El programa está dirigido específicamente a los laboratorios de inmunogenética de trasplantes de órganos inscritos a la Red Nacional de Donación y Trasplantes mediante las instituciones prestadoras de servicios de salud trasplantadoras. Estos laboratorios se catalogan como de alta complejidad debido al personal altamente calificado y capacitado, su tecnología de punta e infraestructura adecuada para llevar a cabo análisis básicos (como la determinación del grupo sanguíneo) hasta procedimientos más complejos (como el análisis de anticuerpos anti-HLA), fundamentales para la categorización inmunológica de los receptores, y la selección óptima de donantes y receptores para la asignación de órganos ^1^.

Mediante esta evaluación, se dispuso de una herramienta para controlar la calidad de los resultados analíticos de estos laboratorios, lo que permite identificar problemas y ejecutar acciones de mejora continua sobre ellos. De esta manera, se garantiza que los resultados emitidos por los laboratorios de la Red de Donación y Trasplantes sean confiables.

Este artículo tiene como objetivo analizar los resultados de la evaluación del desempeño de los laboratorios que practican las pruebas inmunológicas para trasplante en Colombia desde su implementación y a lo largo del tiempo, desde el 2014 hasta el 2023.

## Materiales y métodos

### Diseño del programa

Las instituciones participantes fueron todos los laboratorios clínicos de inmunogenética de trasplantes inscritos cada año a la red por medio de las instituciones trasplantadoras de órganos, por lo que los hallazgos son de alcance nacional. Los laboratorios fueron invitados a participar una vez por año en el “Programa de evaluación externa del desempeño para laboratorios de inmunogenética de trasplantes” y se encuentran ubicados en las ciudades de Bogotá, Medellín, Cali, Bucaramanga, Neiva y Barranquilla. Este estudio incluyó los resultados anuales obtenidos por cada laboratorio de inmunogenética de trasplante de órganos sólidos desde el 2014 hasta el 2023.

Los resultados del programa se basaron en el consenso de los participantes para cada tipo de prueba. Se evaluó el aspecto técnico e interpretativo de cada una para llegar a una puntuación global de rendimiento. Las pruebas inmunológicas evaluadas en cada ejercicio anual fueron las siguientes: tipificación molecular de antígenos leucocitarios humanos (HLA), rastreo e identificación de anticuerpos anti-HLA, panel de antígenos reactivos (PRA) cualitativo y cuantitativo, identificación de anticuerpos anti-HLA por el método de antígenos individuales (*single antigen*) y pruebas cruzadas.

Las muestras para las pruebas (sangre y sueros) suministradas cada año al programa fueron de la misma naturaleza que las utilizadas para las pruebas inmunológicas que se aplican a los donantes de órganos o receptores en la lista de espera. Estas muestras fueron procesadas por el mismo personal especializado que de manera permanente da respuesta a los procesos relacionados con donación de órganos y evaluación de receptores en los laboratorios de inmunogenética en Colombia.

La evaluación anual se hizo de la siguiente forma: para la tipificación de HLA, se analizaron tres muestras y en cada una se evaluaron ocho alelos. Se asignó una calificación para cada muestra y, posteriormente, un resultado del desempeño general del laboratorio en la prueba de tipificación de HLA. Los resultados se evaluaron calculando el grado de concordancia de cada muestra con el consenso de los laboratorios participantes mediante el coeficiente kappa de Cohen para varias categorías. Este índice es una medida estadística más sólida que el porcentaje de concordancia, ya que ajusta el efecto del azar en la proporción observada ^4^. Los valores del coeficiente más cercanos a uno indican mayor grado de concordancia, mientras que los más alejados muestran menor concordancia.

A lo largo de los años, el PRA cualitativo y las pruebas cruzadas también se evaluaron por concordancia mediante el coeficiente kappa de Cohen, mientras que la prueba PRA cuantitativa se evaluó mediante consenso con los resultados de todos los laboratorios participantes calculando la desviación estándar (DE) y el coeficiente de variación (CV) de los resultados de todos los laboratorios participantes. La calificación de cada muestra se asignó teniendo en cuenta la ubicación de su valor según la desviación estándar como «buena»: datos entre +1 y -1 DE; «aceptable»: datos entre +2 y -2 DE e «inaceptable»: datos entre +3 y -3 DE o más.

La prueba de antígenos individuales se evaluó mediante asignación alélica y porcentaje de concordancia. Se calculo un promedio para obtener la calificación de la siguiente forma: «bueno»: 81 a 100 %, «aceptable»: 50 a 80 % e «inaceptable»: menor del 50 %. Después de la evaluación de las tres muestras enviadas en cada ejercicio anual, la calificación final del desempeño de cada prueba se determinó según lo establecido en el cuadro 1.

**Cuadro 1.** Escala de calificación por pruebas del “Programa de evaluación externa del desempeño para laboratorios de inmunogenética de trasplantes”

Grado de desempeño	Pruebas				
	HLA	PRA cualitativo	PRA cuantitativo	Antígenos individuales (%)	Pruebas cruzadas
Bueno	2,91 -3,0	0,61 -1,0	5,1 -6,0	81 - 100	0,61 - 1,0
Aceptable	2,51 -2,9	0,40 - 0,60	4,0-5,0	50-80	0,40 - 0,60
Inaceptable	<2,5	<0,40	<4,0	<50	<0,40
Observación	Se suman los tres índices kappa.	Se reagrupan las categorías del índice kappa.	Suma	Promedio	Se reagrupan las categorías del índice kappa.

Fuente: Programa evaluación externa del desempeño para laboratorios de inmunogenética de trasplantes, 2014-2023

### Análisis estadístico

Los datos se obtuvieron de los resultados del programa conducido por el Instituto Nacional de Salud desde el 2014. Los resultados de las pruebas para cada laboratorio se clasificaron en tres categorías ordinales de calificación, rotuladas como «buena», «aceptable» o «inaceptable», con base en la concordancia de los resultados entre los laboratorios participantes, según el diseño del programa. Se calcularon los porcentajes para cada categoría de calificación por prueba y por año. Se creó la variable «todas» que contenía todos los resultados de las pruebas anuales para medir el desempeño global de los laboratorios participantes. Debido a diferencias en la metodología usada por las cinco pruebas inmunológicas consideradas, se asumió que había independencia en sus resultados, aunque un laboratorio hiciera más de una de ellas.

Se evaluó el supuesto de normalidad en los valores porcentuales del decenio analizado mediante la prueba de Shapiro-Wilk. La categoría «buena» se consideró como ideal de calificación para cada laboratorio y prueba en este sistema. Se establecieron valores puntuales predichos y sus intervalos de confianza del 95 % (1C). Los valores por debajo del límite inferior del intervalo se clasificaron como de menor desempeño al esperado en ese año específico (indicando un menor porcentaje de laboratorios con «buen» desempeño), mientras que aquellos por encima del límite superior denotaron un rendimiento global mayor que el esperado. Los valores dentro del rango del intervalo evidenciaron un desempeño esperado para esa prueba y año.

### Consideraciones éticas

En el presente estudio, se utilizó información secundaria a partir de los resultados obtenidos en el “Programa de evaluación externa del desempeño para laboratorios de inmunogenética de trasplantes”, disponibles para consulta. Las muestras de suero y sangre usadas durante la evaluación hacen parte de los paneles suministrados cada año por entidades contratadas o con convenio con el Instituto, según las especificaciones de cada prueba. Por lo tanto, no se usaron muestras de donantes o receptores de la “Red de donación y trasplantes” que requirieran consentimiento informado.

El “Programa de evaluación externa del desempeño para laboratorios de inmunogenética de trasplantes”tiene una declaración explicita de confidencialidad. A cada laboratorio participante (inscrito oficialmente) se le asigna un código único mediante un número aleatorio. En cada ejercicio anual, este número único de inscripción permite la interacción con los participantes de manera individual y confidencial. Asimismo, el manejo de un código de identificación permite generar informes técnicos o publicaciones sin individualizar los resultados y, así, establecer indicadores de interés para la “Red de donación y trasplantes” y los entes de control a nivel nacional.

Por lo anterior, durante el periodo de estudio, los datos se evaluaron de forma confidencial. Los resultados presentados son globales y pretenden mostrar las tendencias del país, sin entrar en lo particular o al análisis de causas específicas de aquellos fuera de lo esperado.

No se hizo ninguna intervención o modificación intencionada de variables, por lo tanto, este estudio es catalogado como una investigación sin riesgo de acuerdo con la clasificación establecida en la Resolución 8430 de 1993 del Ministerio de Salud ^5^.

## Resultados

Este estudio incluyó una evaluación comparativa anual del desempeño de las pruebas inmunogenéticas efectuadas por el “Programa de evaluación externa del desempeño para laboratorios de inmunogenética de trasplantes” durante 10 años (2014-2023). Para cada año, se contó con una evaluación externa de desempeño, que sumaron un total de 10 evaluaciones en el periodo de estudio.

El programa contó con la participación de todos los laboratorios que realizaban pruebas de inmunogenética para trasplante de órganos sólidos en Colombia. En el 2014, eran 13 laboratorios; en el 2015, eran 12, y del 2016 al 2022, fueron 11. En el 2023, se contó con 10 laboratorios inscritos a la “Red de donación y trasplantes”.

Durante los 10 años de observación, el número de laboratorios participantes fluctuó entre 5 y 12, según el tipo de prueba inmunogenética en el que participaron-HLA, PRA cualitativo, PRA cuantitativo, antígenos individuales y pruebas cruzadas-y el año, lo que dio un total de 415 mediciones de duplas específicas (laboratorio-prueba) y 46 comparaciones de resultados entre laboratorios durante el periodo. El número de laboratorios incluidos en la evaluación varió con cada prueba y año, según la capacidad técnica disponible.

**Figura 1.**
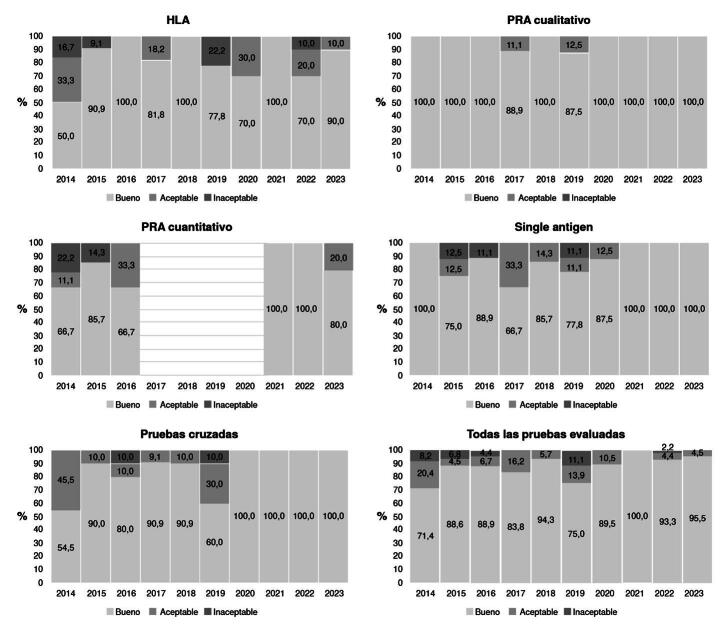
Resultados del “Programa de evaluación externa del desempeño para laboratorios de inmunogenética de trasplantes” por calificación, tipo de prueba y año Nota: Lacalificación de rendimiento «bueno» es la concordancia deseable en las pruebas entre laboratorios y su complemento es la suma de «aceptable» e «inaceptable».

Los resultados comparativos entre los laboratorios se calificaron como «buenos», «aceptables» o «inaceptables» para cada una de las pruebas evaluadas y el consolidado global. Su distribución por tipo de prueba y año se muestra en la figura 1.

Fuente: Programa de evaluación externa del desempeño para laboratorios de inmunogenética de trasplantes, 2014-2023

El rendimiento fue calificado como «bueno» en menor proporción durante el primer año de evaluación, en especial, en el HLA, el PRA cuantitativo y las pruebas cruzadas. Solo en los dos primeros años, hubo laboratorios con rendimiento «inaceptable». La mejor concordancia entre laboratorios se observó para el PRA cualitativo, ya que todos se calificaron como «bueno» en 8 de los 10 años observados; y para los otros dos años, «aceptable» fue la menor calificación obtenida por los laboratorios. En el caso del PRA cuantitativo, entre el 2017 y el 2020, no se encontraron datos para este ítem debido a que no hubo laboratorios suficientes para lograr el consenso en esta prueba. Sin embargo, en el último trienio, la concordancia fue considerada como «buena» en todos los laboratorios. En el 2023, solo un laboratorio fue calificado como «aceptable», sin encontrarse ninguno con rendimiento «inaceptable».

La prueba de antígenos individuales tuvo tres años de rendimiento «inaceptable» en algunos laboratorios, pero en los últimos tres años (2021-2023) de observación, todos se calificaron como «bueno». Mientras que, en el caso de las pruebas cruzadas durante los últimos cuatro años, todos los laboratorios se calificaron como «bueno».

**Figura 2.**
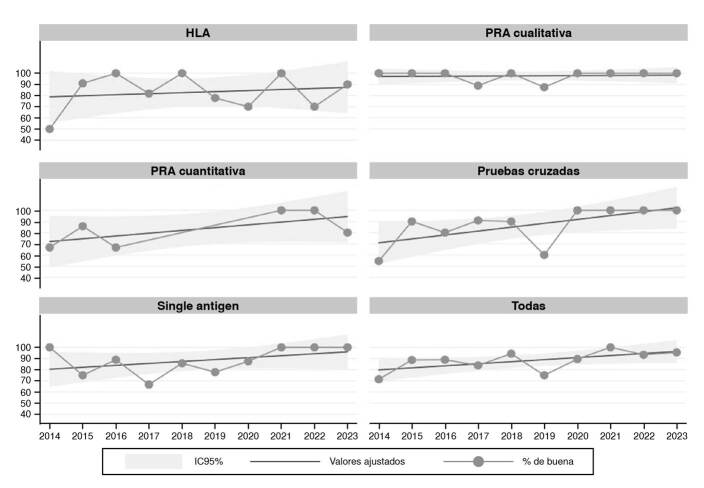
Valores observados, predichos e intervalos de confianza en la evaluación del desempeño entre laboratorios clasificada como "buena” por tipo de prueba y año Nota: El área sombreada es el intervalo de confianza del 95 %. Los puntos por encima del límite superior indican un rendimiento mayor que el esperado, mientras que los situados por debajo del límite inferior son aquellos con rendimiento menor que el esperado.

La figura 2 muestra los valores observados, predichos y los intervalos de confianza para las proporciones resultantes de la evaluación del desempeño entre laboratorios clasificados como «bueno», por tipo de prueba y año. El área sombreada es el intervalo de confianza del 95 %. Los puntos por fuera del límite superior del intervalo de confianza indican un rendimiento por encima del esperado, como se observó para el HLA en el 2018 y para los antígenos individuales en el 2014. En el caso del PRA cualitativo, en la mayoría de los años de observación (8 de 10), el desempeño de los laboratorios se clasificó como «bueno» en todos los casos. Por su parte, aquellos valores por debajo del límite inferior del IC_95%_, como HLA (2014), PRA cualitativo (2017-2019), pruebas cruzadas (2019) y antígenos individuales (2017-2019), indicaron un menor porcentaje de laboratorios con desempeño «bueno» menor que el esperado.

Fuente: Programa de evaluación externa del desempeño para laboratorios de inmunogenética de trasplantes, 2014-2023

Al unificar las calificaciones en las pruebas («Todas», figura 2), se observa que solo en el 2014 y el 2019 se espera que las pruebas sean satisfactorias. Llama la atención el año 2021 dado que todos los laboratorios tuvieron un desempeño «bueno» en todas las pruebas evaluadas. Todas las pruebas alcanzaron una calificación de «bueno» en su desempeño, al menos, en uno de los tres últimos años, pero es notable el desempeño de los laboratorios en todo el periodo para el PRA cualitativo y, en los últimos tres y cuatro años de observación, para los antígenos individuales y las pruebas cruzadas, respectivamente.

En las pruebas de HLA, se notó un incremento notable en el porcentaje de acierto desde el Inicio del estudio, alcanzándose la calificación de «bueno» en la mayoría de los laboratorios evaluados hacia el final del periodo de estudio. Este resultado es indicativo de una estandarización efectiva en los procedimientos de tipificación de HLA, y refleja la importancia de la experiencia acumulada y el intercambio de conocimientos entre los laboratorios.

Las pruebas PRA cualitativa y cuantitativa, fundamentales para la detección de anticuerpos que pueden influir en el éxito del trasplante, también mostraron una mejora en su calificación a lo largo de los años. Este avance puede atribuirse a la implementación de tecnologías más sensibles y específicas, así como a una mayor comprensión de su impacto en la selección de donantes y receptores compatibles.

## Discusión

En la posición cromosómica 6p21 del genoma humano, se localizan los genes del HLA, que se expresa de manera codominante para la presentación de antígenos. Los genes HLA se han asociado con diversas entidades clínicas, como infecciones, cáncer o enfermedades autoinmunitarias; se ha demostrado que desempeñan un papel protagónico en los procedimientos de trasplante, ya que ciertas moléculas de HLA pueden inducir una respuesta inmunológica particular y provocar el rechazo del órgano ^6^^-^^12^.

Por lo anterior, los sistemas estandarizados por las organizaciones de donación y trasplante-incluido el de Colombia-se fundamentan en la tipificación del HLA y la detección de anticuerpos anti-HLA para la asignación de órganos para trasplantes de riñón, corazón y pulmón. Estas dos pruebas constituyen uno de los factores con mayor peso para establecer la mayor compatibilidad entre donante y receptor, por ejemplo, en el trasplante renal, en el cual existe la mayor cantidad de pacientes en lista de espera ^13^^-^^16^.

La tipificación del HLA y la detección de anticuerpos anti-HLA son cruciales para lograr un trasplante compatible con menor riesgo de rechazo ^6^^,^^12^. En este sentido, el papel que juegan los laboratorios de inmunogenética de trasplantes es fundamental para garantizar programas eficientes y con mejores resultados a largo plazo. Así, el control del desempeño, mediante los programas de evaluación enfocados en la mejora continua, brinda herramientas para identificar las desviaciones y el inicio de acciones inmediatas sobre las mismas. Esta evaluación asegura un desempeño comparable entre los laboratorios, y proporciona confianza en los sistemas de donación y trasplante, en los cuales los principales beneficiados son los pacientes.

La asignación de trasplantes renales en Colombia, según los criterios nacionales definidos por el Instituto Nacional de Salud, incluye como requisitos iniciales la tipificación de HLA de donantes y receptores con técnicas de mediana resolución para los locus HLA-A, HLA-B, HLA-DR y HLA-DQ. Asimismo, el estudio previo al trasplante para cada paciente debe incluir las pruebas de PRA cualitativa y cuantitativa, la detección de antígenos individuales y la determinación de incompatibilidades inaceptables. El seguimiento del riesgo inmunológico se debe realizar mientras los receptores estén activos en la lista de espera ^13^. De acuerdo con estos criterios, la asignación de órganos -un proceso crítico del cual se derivan algunos resultados clínicos del receptor del trasplante-depende de resultados fiables y oportunos provenientes de los laboratorios de inmunogenética. La calidad de estos resultados debe incluir controles internos y externos adecuados, como los que ejecuta el “Programa de evaluación externa del desempeño para laboratorios de inmunogenética de trasplantes”.

Como parte de sus funciones como coordinador nacional de la “Red de donación y trasplante” en Colombia, el Instituto Nacional de Salud propende por ejecutar acciones encaminadas a mejorar la seguridad y la calidad de los órganos trasplantados en el país; además, es la entidad nacional de referencia encargada de la supervisión de laboratorios en áreas de interés en salud pública mediante diferentes programas de evaluación externa del desempeño ^17^. El Instituto incluyó los laboratorios de inmunogenética de trasplantes como actores esenciales en los procesos de la “Red de donación y trasplante”. Por lo tanto, estos laboratorios están sujetos a las acciones de control y mejora continua, implementándose dos acciones estratégicas para tal fin: 1) la documentación específica para estos laboratorios en los estándares de calidad exigidos por la Red de donación y trasplantes, los cuales se aplican mediante el programa de auditoría externa de la Red y 2) la realización del Programa de evaluación correspondiente.

El Programa fue diseñado específicamente para Colombia, ya que los paneles de control deben simular las condiciones de calidad de las muestras de un donante o un receptor para trasplante y la logística de su traslado.

Para algunos analitos de la prueba, los paneles están constituidos por muestras frescas que requieren una distribución rápida, preferiblemente dentro de las 24 horas ^18^^,^^19^. Por lo tanto, cada panel debe organizarse y trasladarse en un mismo día a cada una de las ciudades en donde se ubican los laboratorios participantes. De esta forma, el programa verifica la disponibilidad ininterrumpida y la capacidad del laboratorio para procesar este tipo de muestras en cualquier hora del día, como normalmente pasa en los procesos de donación y trasplante de órganos.

Diversos autores indican que las pruebas de evaluación externa del desempeño en la tipificación de HLA, contribuyen a evaluar la reproducibilidad, precisión y confiabilidad de los resultados emitidos por los laboratorios participantes, y a la gran calidad en los procesos relacionados con el trasplante ^20^^-^^22^. En Colombia, el “Programa de evaluación externa del desempeño para laboratorios de inmunogenética de trasplantes” aplicado directamente por el Instituto, como entidad pública nacional y coordinador de donación y trasplantes, ha permitido evaluar la calidad de las pruebas inmunológicas usadas en procesos críticos, como la asignación de órganos. Asimismo, el programa ha permitido desarrollar acciones preventivas y correctivas cuando ha habido lugar a ello, promoviendo una confiabilidad creciente en los procesos.

En otros programas de evaluación externa, algunos autores reportan una concordancia cercana al 100 % en tipificaciones de HLA ^20^^,^^22^. En Colombia, los resultados obtenidos en el presente estudio indican que, si bien en los primeros años el desempeño no fue óptimo para todos los laboratorios participantes, en los últimos años, ha habido una mejoría con una buena calificación del desempeño que refleja gran concordancia, lo cual justifica la necesidad de continuar desarrollando esta estrategia de evaluación y control.

En los casos de laboratorios con desempeño inaceptable en alguna prueba evaluada, el Instituto llevó a cabo procesos de asistencia técnica, y propendió a la formulación y el cumplimiento de planes de mejora.

Respecto a los programas de evaluación externa del desempeño de pruebas cruzadas y detección de anticuerpos anti-HLA, según lo reportado por Duquesnoy *et al*., en los primeros años de implementación de este tipo de programas en Estados Unidos ^22^, se obtuvieron resultados diversos con concordancias menores que las de HLA y con diferencias importantes según las técnicas utilizadas. En Colombia, este es el primer estudio que muestra los resultados de diez años de un programa de evaluación externa del desempeño aplicado a laboratorios de inmunogenética de trasplantes, en el cual, los niveles de concordancia de las pruebas cruzadas y de detección de anticuerpos anti-HLA (PRA) han sido variables; sin embargo, en los últimos tres años ha habido una tendencia creciente a la mejoría del desempeño en estas pruebas.

Por otro lado, la presencia de anticuerpos específicos del donante contra moléculas de HLA, tiene efecto en la supervivencia del injerto después del trasplante de órganos ^23^^-^^26^. Por esta razón, la calidad con la que se practiquen este tipo de pruebas diagnósticas impacta en los resultados clínicos de los pacientes, por lo que su evaluación ocupa un lugar importante en los procesos de calidad de un sistema de donación y trasplante ^20^^,^^22^.

Los resultados del programa de Colombia reflejan que existe mayor variabilidad en las pruebas de PRA cuantitativo y antígenos individuales, en comparación con la tipificación de HLA y el PRA cualitativo, estas últimas con una mejor calificación de desempeño. Esto puede deberse a que las pruebas de cuantificación de anticuerpos, así como las de identificación de alelos específicos (antígenos individuales), utilizan algunos parámetros generalmente no estandarizados, como puntos de corte y valor medio de fluorescencia que, además, dependen de los conocimientos teóricos y prácticos del programa de trasplantes y del laboratorio ^27^^-^^30^.

La desviación de un resultado o de su interpretación tiene implicaciones importantes en los resultados clínicos del trasplante de un paciente. Por esta razón, a pesar de las diferencias que puedan existir entre las técnicas, se debe procurar alcanzar la mayor estandarización posible. Por otro lado, el trasplante proviene de un esfuerzo de los programas de donación por obtener “más órganos”, en un escenario mundial donde la demanda de órganos supera los donantes existentes, tal como ocurre en Colombia ^15^. Esto conlleva que los programas de trasplante busquen lograr mejores estándares de calidad en sus procesos para garantizar mejores resultados en términos de supervivencia y menos complicaciones en los pacientes. Las pruebas inmunológicas juegan un papel muy importante en el proceso de trasplante desde la inscripción adecuada de un paciente a la lista de espera, la asignación de órganos y el trasplante, hasta la etapa de seguimiento después del trasplante.

La experiencia presentada demuestra la importancia que tienen las acciones de control y evaluación de las entidades que conforman la “Red de donación y trasplante” y, en general, de cualquier sistema de este tipo. El intercambio de órganos en estos sistemas requiere estandarizar al máximo procesos comunes, independientemente de la entidad que los ejecute como, en este caso, los laboratorios de inmunogenética. Estas entidades son pieza clave en la donación y el trasplante; por esto, requieren ejecutar acciones de control de calidad a sus pruebas, dado el gran impacto clínico que tienen en los pacientes en espera de un trasplante y las implicaciones de sus desviaciones o la heterogeneidad de los resultados ^19^^,^^22^.

EI “Programa de evaluación externa del desempeño para laboratorios de inmunogenética de trasplantes” es una herramienta para el control de calidad de los resultados analíticos, dado el impacto de estas pruebas en las diferentes etapas del proceso de trasplante, como la compatibilidad para la asignación de órganos, las terapias de desensibilización, la categorización inmunológica de receptores en lista de espera y la definición de alelos inaceptables, entre otras. Las desviaciones o dificultades en la interpretación de las pruebas pueden afectar los resultados clínicos, por lo que se debe mantener un programa de evaluación externa que continúe incentivando la mejora y la fiabilidad de los resultados emitidos en beneficio de los pacientes.

Todas las pruebas muestran un desempeño mejor en el último trienio. El cambio más notable es el de la tipificación de HLA, pues los resultados analizados en el presente estudio reflejan una mejoría a través del tiempo. Estos hallazgos ratifican la necesidad de continuar con este Programa con el fin de lograr una mayor estandarización.

Se recomienda una mayor articulación entre los especialistas de los laboratorios de inmunogenética y los programas de trasplante, mediante su participación en las juntas de trasplante para decidir sobre cada caso que ingresa a lista de espera o en reuniones periódicas en las que se discutan los casos con características inmunológicas especiales.

La aplicación de este tipo de controles de calidad para la mejora continua de las pruebas de inmunogenética de trasplantes es beneficiosa para los procesos de la Red de donación y trasplantes, ya que los laboratorios de inmunogenética deben contar con oportunidad, disponibilidad, manejo de muestras e interpretación de resultados, para atender las condiciones de urgencia que implica un proceso de donación y trasplante, de intercambio de órganos o de traslado de pacientes en lista de espera.
